# Risk Factors for Cognitive Decline in Older Adults in Puerto Rico: Assessing Bias From Sample Attrition

**DOI:** 10.1093/geroni/igab046.709

**Published:** 2021-12-17

**Authors:** Brian Downer, Caitlin Pope, Tyler Bell, Sadaf Milani, Ross Andel, Michael Crowe

**Affiliations:** 1 University of Texas Medical Branch, Galveston, Texas, United States; 2 University of Kentucky, Lexington, Kentucky, United States; 3 University of California San Diego, La Jolla, California, United States; 4 University of South Florida, Tampa, Florida, United States; 5 University of Alabama at Birmingham, Birmingham, Alabama, United States

## Abstract

Many risk factors for cognitive decline are associated with mortality and are common among older adults who cannot complete a survey interview. Our objective was to compare analyses of risk factors for cognitive decline among older adults in Puerto Rico with and without accounting for sample attrition. Data came from the Puerto Rican Elderly: Health Conditions Study. Our sample included 3,437 participants interviewed in 2002/03. Cognitive function was measured using the Mini-Mental Caban (MMC). The outcome was the change in MMC score between 2002/03 and 2006/07. Logistic regression was used to estimate inverse probability weights for being interviewed in 2006/07 (n=3,028) and completing the MMC at follow-up (n=2,601). Linear regression models were used to assess the association between stroke, hypertension, diabetes, smoking status, and cognitive decline with and without the IPWs. In the unweighted analysis, stroke was associated with a significantly greater decline in cognition (b=-0.62, standard error [SE]=0.30, p=0.04). Hypertension (b=-0.02, SE=0.12, p=0.84), diabetes (b=-0.22, SE=0.13, p=0.10) and being a current (b=0.05, SE=0.22, p=0.84) or former smoker (b=0.05, SE=0.14, 0.74) were not associated with cognitive decline in the unweighted analysis. The results were similar when including the IPW for mortality (stroke b=-0.63; hypertension b=-0.03; diabetes: b=-0.20; current smoker: b=0.08; former smoker: b=0.07) and having completed the MMC at follow-up (stroke b=-0.58; hypertension b=-0.03; diabetes: b=-0.20; current smoker: b=0.03; former smoker: b=0.09). These findings indicate that stroke is a risk factor for cognitive decline among older Puerto Rican adults even after accounting for selective attrition.

